# Initial acidic media promotes quiescence entry in *Saccharomyces cerevisiae*

**DOI:** 10.17912/micropub.biology.001071

**Published:** 2024-02-24

**Authors:** Alison Greenlaw, Rachel Dell, Toshio Tsukiyama

**Affiliations:** 1 Basic Sciences Division, Fred Hutch Cancer Center, Seattle, Washington, United States

## Abstract

Quiescence is a conserved cellular state wherein cells cease proliferation and remain poised to re-enter the cell cycle when conditions are appropriate. Budding yeast is a powerful model for studying cellular quiescence. In this work, we demonstrate that the pH of the YPD media strongly affects quiescence entry efficiency in *Saccharomyces cerevisiae.* Adjusting the initial media pH to 5.5 significantly improves quiescence entry efficiency compared to unadjusted YPD media. Thermotolerance of the produced quiescence yeast are similar, suggesting the media pH influences the quantity of quiescent cells more than quality of quiescence reached.

**Figure 1. Initial acidic pH promotes quiescence entry:**
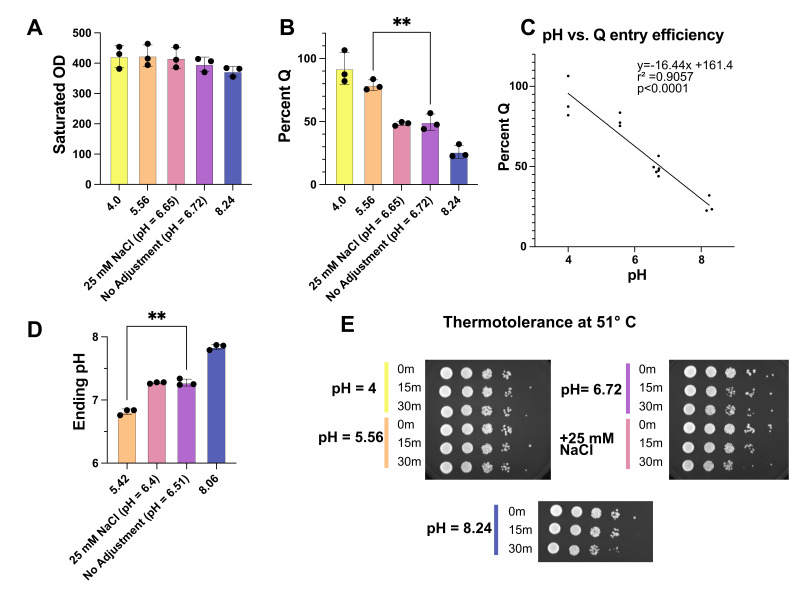
( **A** ) Bar graph showing total saturated OD _660_ of yeast produced by each media condition. Points represent each replicate and bars are standard deviation. Using pair t-test, difference is not significant. ( **B** ) Bar graph showing percent quiescent cells produced by each media condition. Points represent each replicate and bars are standard deviation. Using pair t-test, difference between 5.56 and 6.72 is significant (** = p < 0.001). ( **C** ) Scatter plot comparing media pH to percent quiescent. Linear regression analysis performed using Prism. ( **D** ) Bar graph showing ending pH of 7-day saturated culture. Using pair t-test, difference between 5.42 and 6.51 is significant (** = p < 0.001). ( **E** ) Spot test of quiescent cell thermotolerance at 51 °C.

## Description

Quiescence is a conserved process in which cells exit from the mitotic cell cycle for long term survival in eukaryotes. *Saccharomyces cerevisiae* (hereafter yeast) have been leveraged as an effective system to study quiescence, since large quantities of quiescent cells can be readily purified from stationary phase culture (Allen et al., 2006) . When quiescent cells are purified using the Percoll gradient method, quiescent (heavy) and non-quiescent (light) fractions are separated, and the percent of cells which enter quiescence can be measured.

We have found that quiescence entry efficiency of the same yeast strain is variable over time, especially dependent upon the batch of YPD (Yeast extract Peptone Dextrose) media used. This was a logistical hurdle, as mutant strains with low quiescence entry efficiency had to be grown and purified at large scale to ensure sufficient quiescent cell yield for genomic and biochemical experiments. Additionally, the necessary scale of the cultures required was difficult to predict due to the variability of quiescence entry. One aspect of the media that was uncontrolled in our laboratory was the pH. Standard recipes for liquid YPD do not include adjusting media pH. Acidic pH has been demonstrated to increase glycerol production (Yalcin & Yesim Ozbas, 2008) and basic pH has been shown to inhibit fermentation and respiration in yeast (Peña et al., 2015). In an industrial strain of yeast, pH 5.5 has been reported as the optimal pH for ethanol production (Narendranath & Power, 2005) . We therefore wondered if media pH might influence quiescence entry efficiency.

To test this possibility, three strains in five different pH-adjusted YPD media were grown. Four pH measurements were used: 4.0, 5.56, 6.72 (no adjustment) and 8.24. Since acetic acid is toxic to yeast and promotes aging (Burtner et al., 2009; Murakami et al., 2011) , an inorganic acid (hydrochloric acid) and base (sodium hydroxide) were used to test the effect of media pH on quiescence entry. As an additional control, we added low concentration sodium chloride to the 5 ^th^ YPD, to test whether the pH, or the addition of chloride or sodium ions was responsible for the observed changes to quiescence entry.

Yeast grown in all five batches of media saturated at ~30 OD _660_ per mL, producing approximately 400 total OD _660_ ( Figure 1A ). Saturated density is essentially equivalent for all five media, with a slight but statistically insignificant decrease for the basic pH media (p = .1410, paired t-test comparing pH = 5.56 and pH = 8.24). In contrast, pH dramatically changes the fraction of cells which are quiescent ( Figure 1B ). YPD with pH 4.0 results in an approximately 90% quiescence entry efficiency, compared to approximately 25% efficiency at pH 8.24. The addition of 25 mM sodium chloride did not significantly change quiescence entry efficiency compared to unadjusted YPD, demonstrating that pH, not salt ions, is the most relevant variable (p = .7822, paired t-test comparing 25 mM NaCl to unadjusted YPD). Quiescence entry efficiency and pH are highly correlated between pH 4 and pH 8.24 (r ^2^ = .9057) ( Figure 1C ). Using simple linear regression, the estimated slope is -16.44, and it is significantly non-zero (p<0.0001). Based on these results, we suggest using YPD media with pH 5.5. We find this results in robust quiescent cell yields and reduces batch to batch variability.

We wondered if the initial pH of media changed the pH of the final saturated culture after seven days. We find that acidic initial pH results in an acidic final pH (1D). The final pH is significantly lower in pH 5.42 than in pH 6.51 (p = 0.0034, paired t-test). Additionally, change in pH between the initial and final values depends upon the starting pH. For initial pH 5.42 the culture becomes more basic over the period of seven days, with a mean final pH of 6.81 (SD= 0.04). This same pattern holds for the unadjusted media and 25 mM NaCl, with a mean final pH of 7.28 and 7.27 respectively (SD _unadjusted_ = 0.06 and SD _NaCl_ = 0.01). However, when the initial pH is basic in the case of pH 8.06, the culture becomes very slightly more acidic (mean final pH = 7.84, SD = .044). Further work will be necessary to understand what metabolic processes produce these pH changes, and how they are affected by initial pH. Whether final pH of the culture is incidental, or a driving factor of altered quiescence entry is unclear. However, since initial pH can easily be controlled in the laboratory, we chose to focus on this aspect.

To test if the cells produced were truly quiescent, a thermotolerance assay was performed. Quiescence yeast have improved thermotolerance, and can survive heat shock at 51 °C (Allen et al., 2006; Klosinska et al., 2011) . Thermotolerance is relatively similar regardless of media pH ( Figure 1E ). Although the difference is very minor, pH 4.0 and 5.56 seem to survive slightly better than pH 6.72 and 8.24 after 30 minutes at 51 °C. This suggests that the increased quiescence entry yields from the media with pH 4.0 and 5.56 are the result of additional *bona fide* quiescent cells.

In contrast to these results, previous reports demonstrated that acetic acid is toxic to yeast, and low pH has been shown to reduce chronological lifespan (Burtner et al., 2009; Murakami et al., 2011) . These experiments were performed in auxotrophic strains, which have poor longevity and are not appropriate for studying quiescence (Boer et al., 2008; Breeden & Tsukiyama, 2022) , whereas we used prototrophic strains in our work. Additionally, BY4741 was used for some experiments, which does not enter quiescence (Miles et al., 2023) . Therefore, the apparent differences in the effects of low pH media between previous reports and our results are likely due, at least in part, to the differences in strain backgrounds.

## Methods

Making media of different pH

A 2-liter batch YEP was made (1% yeast extract, 2% peptone) and split into five 375 mL aliquots. pH was then adjusted with either HCl (for pH 4 and 5.5) or NaOH (for pH 8.5). As an additional control, NaCl was added at 25 mM to an aliquot. This concentration was determined based on the amount of HCl added to the pH 4 aliquot. The amount of acid, base or salt added was measured, and a remaining volume of water was added to so that 10 mL of additional volume were added to each aliquot. For the unadjusted control, 10 mL of water were added. All 5 media were autoclaved for 1 hour at 120˚C. Glucose was added to a final concentration of 2%, and the pH was re-measured. See below table for additional details:

**Table:** 

**Added to 375 mL YEP**	**pH before autoclaving**	**pH after autoclaving and adding glucose to 2%**
10 ml 1N HCl	4.0	4.0
2.25 ml 1N HCl + 7.75 mL water	5.46	5.56
10 mL water	Not measured	6.65
6.66 mL 1.5 M NaCl + 3.33 mL water	Not measured	6.72
7 mL 1N NaOH + 3 mL water	8.46	8.24

Quiescent yeast growth, and purification

10 µl of yeast overnight culture from unadjusted YPD was added to each flask and grown for 7 days to saturation at 30 °C on a shaker set to 180 rpm. All cultures were grown in 125 mL flasks with 12.5 mL of media. Saturated density was easured by diluting saturated culture 1/100 ^th^ in YPD and taking OD _660_ using a spectrophotometer. Total saturation density was calculated by multiplying OD _660_ by 100 for dilution and 12.5 for total volume.

Quiescent cells were purified using the Percoll density gradient method as previous described (Allen et al., 2006) . Briefly, Percoll and 1.5 M NaCl were combined in an 11:1 ratio, and 12.5 mL of this mixture was added to a gradient column. Columns were centrifuged at 10,000 rpm for 15 minutes at 4 °C to create a gradient. Saturated yeast cells were harvested and resuspended in 3 mL of sterile double distilled water, then the water and yeast mixture was carefully layered over the column. Columns were centrifuged for 1 hour at 1,200 rpm at 4 °C. The top of the column was discarded and the bottom ~5 mL section was taken as the quiescent fraction. The quiescent fraction was washed with sterile double distilled water twice and resuspended in 9 mL of sterile double distilled water. Final volume of quiescent fraction was measured using a 10 mL serological pipet.

Quiescent cell yield was taken by diluting quiescent fraction 1/100 ^th^ in water and taking OD _660_ using a spectrophotometer. OD _660_ was multiplied by 100 for dilution and by the final volume for total quiescent cell yield. Of note, quiescence entry percent was calculated from the total OD _660_ of the saturated culture and total OD _660_ of the quiescent cells. The saturated culture was in YPD and blanked against YPD, while final Q fraction was in water and blanked against water. In one case this led to the total quiescent cell yield being calculated as greater than 100 percent.

Final pH measurements

The same media were used to measure final pH as in all previous experiments. Since this experiment was performed several months after, pH was remeasured. 4.0 was excluded as the media had become contaminated. Cultures were inoculated and grown as described above. Final pH was measured after pelleting the cells 7 days after inoculation. The media were filter-sterilized before measurement; filters were rinsed with sterile water between media samples, to avoid cross-contamination. Standardization of the pH meter and measurement were kept consistent throughout.

Thermotolerance assay

Heat shock was performed at 51 °C based on previous reports in quiescent yeast (Allen et al., 2006; Klosinska et al., 2011) . Quiescent yeast at a concentration 1 OD _660_ per mL were aliquoted into microcentrifuge tubes. Two aliquots were placed in hot water bath at 51 °C for 15 or 30 minutes. An aliquot was kept on the bench as a no heat control. Yeast were serially diluted 1:10 4 times and then 2 µl of each was plated on a YPD plate. The plates were grown at room temperatures for 3 days and then imaged using Biorad Universal Hood II Gel Doc XR System with epi-white light. Images were cropped for the figure to remove empty space, but no other image adjustments were performed.

Statistical Analysis

All experiments were performed in biological triplicate with independently created stains. Graphing and statistical analysis were performed in Prism (version 10). Paired t-test was used to compare between columns in figure 1A, B and D . Simple linear regression was used to generate the trend line in figure 1C .

## Reagents

**Table:** 

**Strain**	**Genotype**	**Available from**
yTT5781	*MATa RAD5+ can1-100*	Tsukiyama Lab
yTT5782	*MATa RAD5+ can1-100*	Tsukiyama Lab
yTT5779	*MAT* α *RAD5+ can1-100*	Tsukiyama Lab

**Table:** 

**Name**	**Supplier**	**Catalogue number**
Dextrose (D-Glucose), Anhydrous (Granular Powder/Certified ACS)	Thermo Fisher	D16-10
Gibco™ Bacto™ Peptone	Thermo Fisher	211820
Gibco™ Bacto™ Yeast Extract	Thermo Fisher	212750
Hydrochloric Acid	Thermo Fisher	A481-212
Sodium Hydroxide	Sigma Aldrich	S8045
Sodium Chloride	Thermo Fisher	BP538-10
Percoll PLUS	Cytiva	17544501
